# An *in situ* fluorine and *ex situ* titanium two-step co-doping strategy for efficient solar water splitting by hematite photoanodes†

**DOI:** 10.1039/d2na00029f

**Published:** 2022-02-12

**Authors:** Kyoungwoong Kang, Hemin Zhang, Jeong Hun Kim, Woo Jin Byun, Jae Sung Lee

**Affiliations:** School of Energy and Chemical Engineering, Ulsan National Institute of Science and Technology (UNIST) 50 UNIST-gil Ulsan 44919 Republic of Korea jlee1234@unist.ac.kr; College of Materials Science and Engineering, Sichuan University Chengdu 610065 China hmzhang@scu.edu.cn

## Abstract

A unique two-step co-doping strategy of *in situ* fluorine doping followed by *ex situ* titanium doping enhances the performance of the hematite photoanode in photoelectrochemical water splitting much more effectively than single-step co-doping strategies that are either all *in situ* or all *ex situ*. The optimized fluorine, titanium co-doped Fe_2_O_3_ photoanode without any cocatalyst achieves 1.61 mA cm^−2^ at 1.23 V_RHE_ under 100 mW cm^−2^ solar irradiation, which is ∼2 and 3 times those of titanium or fluorine singly-doped Fe_2_O_3_ photoanodes, respectively. The promotional effect is attributed to the synergy of the two dopants, in which the doped fluorine anion substitutes oxygen of Fe_2_O_3_ to increase the positive charges of iron sites, while the doped titanium cation substitutes iron to increase free electrons. Moreover, excess titanium on the surface suppresses the drain of *in situ* doped fluorine and agglomeration of hematite during the high-temperature annealing process, and passivates the surface trap states to further promote the synergy effects of the two dopants.

## Abstract 2

A novel two-step co-doping strategy of *in situ* F^−^ anion, followed by *ex situ* Ti^4+^ cation promotes the hematite photoanode for water splitting much more effectively than single-step co-doping strategies that are either all *in situ* or all *ex situ*.

## Graphical abstract

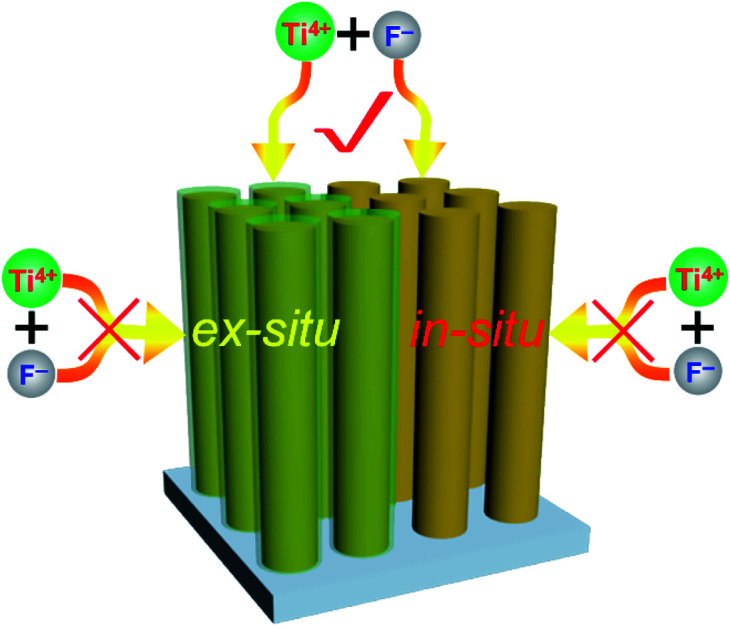

## 1. Introduction

Water splitting with sunlight has attracted great research interest in recent decades as an ideal route to renewable and storable hydrogen (“green hydrogen”).^1–3^ Among several options for solar hydrogen production, the photoelectrochemical (PEC) cell has been regarded as a promising technology for practical applications because it is more efficient than the particulate photocatalytic cell, and cheaper than the photovoltaic–electrolyzer cell.^4^ The performance of a PEC cell is determined by its photoelectrode material, which requires a narrow band gap, appropriate conduction and valence band positions, low cost, and high operational stability. Despite intensive research and many technical advances in the last few decades, no photoelectrode material has met all of these requirements.^5^

Hematite (α-Fe_2_O_3_) is one of the most extensively studied photoanode materials because of its highly desirable properties including ample visible-light absorption, good stability in aqueous solutions, non-toxicity, earth-abundance, and low cost. With its band gap of 2.1 eV, an ideal hematite photoanode can generate a photocurrent density as high as 12.6 mA cm^−2^ under solar irradiation of 100 mW cm^−2^ (AM 1.5G) or a solar to hydrogen (STH) conversion efficiency of 15.5%.^6^ However, the state-of-the-art hematite photoanodes reported so far have only achieved less than half of the theoretical limit because of its intrinsically poor optoelectronic properties of short diffusion length of the photogenerated holes (2–4 nm), low electrical conductivity (10^−2^ cm^2^ V^−1^ s^−1^), and slow water oxidation kinetics.^7,8^

A number of modification strategies have been developed to alleviate the shortcomings of hematite such as doping, nanostructuring, hetero-/homo-junctions, surface modifications and co-catalysts.^9–13^ In particular, doping and nanostructuring are the most effective ways to reduce the recombination of photogenerated electrons and holes in the bulk of hematite. Foreign atom doping can increase the charge carrier density to enhance the electrical conductivity of hematite, while nanostructuring can help overcome the problem of its extremely short hole diffusion length.^14^ Hence, doping and nanostructuring in combination could exert a synergistic effect in reduction of bulk recombination.

The most common dopants for hematite are tetravalent metal cations (M^4+^ = Ti^4+^, Sn^4+^, Zr^4+^, Si^4+^, or Pt^4+^), which provide an additional free electron when these M^4+^ ions substitute for Fe^3+^ in the hematite lattice. Recently, fluorine anions were used as an effective dopant by replacing oxygen atoms in metal oxide semiconductors (TiO_2_, WO_3_, and ZnO) including hematite owing to its ionic radius (119 pm) being similar to that of oxygen (126 pm), which resulted in multiple desirable effects – promotion of electron transfer from the localized states to the conduction band, enhanced light harvesting, and decreased band gap.^15–17^ In light of these previous studies, we conjectured that a hematite photoanode co-doped with metal cations and non-metal anions might provide a new path to improved PEC performance. There have been several reports on co-doped Fe_2_O_3_ with different cations,^18–20^ but this type of cation (M^4+^) and anion (F^−^) co-doping of hematite has never been investigated previously.

In this work, we explore the synergistic co-doping effects of the fluorine anion (F^−^) and titanium cation (Ti^4+^) into the hematite lattice according to a unique two-step doping process – *in situ* F^−^-doping during the hydrothermal synthesis of FeOOH nanorods followed by *ex situ* Ti^4+^-doping during FeOOH-to-Fe_2_O_3_ conversion under high-temperature annealing. It is demonstrated that the two-step co-doping process promotes the PEC performance of the hematite photoanode much more effectively than single-step co-doping strategies that are either all *in situ* or all *ex situ*. In the finally obtained F^−^, Ti^4+^-codoped Fe_2_O_3_ nanorods (F,Ti:Fe_2_O_3_), the F^−^ anion substitutes oxygen of Fe_2_O_3_ to enhance the positive charges of the iron sites, while Ti^4+^ substitutes iron to generate more free electrons, thereby both contributing to electrical conductivity and n-type character. In addition, excess titanium remaining on the surface suppressed the drain of *in situ* doped fluorine and agglomeration of hematite during the high-temperature annealing process and passivated the surface trap states to further promote the synergy effects of the two dopants. This beneficial side effect is only possible for our two-step co-doping strategy. As a result, the F,Ti:Fe_2_O_3_ photoanode without any cocatalyst generates a photocurrent density of 1.61 mA cm^−2^ at 1.23 V_RHE_ under 1-sun irradiation, far outperforming those of pristine and singly-doped hematite photoanodes.

## 2. Experimental section

### 2.1. Fabrication of F,Ti:Fe_2_O_3_ nanorods on F:SnO_2_ (FTO) coated glass

Several pieces of FTO (PECTM 8, 6–9 Ω, Pilkington) were ultrasonically cleaned by using a chemical agent (deconex® 11 Universal) solution, ethanol and acetone, respectively, which made the surface sufficiently hydrophilic. The FeOOH nanorods were hydrothermally synthesized on the FTO glass. Briefly, into a 10 mL aqueous solution of 0.15 M FeCl_3_·6H_2_O, and 1 M NaNO_3_ at pH of ∼1.5, FTO glass was immersed and kept in an electric oven at 100 °C for 4 h to obtain a thin yellow film of FeOOH nanorods on FTO. For F-doping, NH_4_F was added in the precursor solution to obtain F:FeOOH nanorods. For Ti-doping, a diluted titanium(iv) chloride solution using 2-methoxyethanol was deposited on FeOOH or F:FeOOH nanorods by spin-coating at 2000 rpm for 20 s. For single-step F and Ti *in situ* co-doping, NH_4_F and titanium chloride were added in the precursor solution to obtain F,Ti:FeOOH nanorods. For single-step *ex situ* co-doping, NH_4_F and titanium butoxide were diluted in 2-methoxyethanol and deposited on the surface of FeOOH nanorods by spin-coating. The FeOOH or F:FeOOH nanorods with/without titanium were heated at 550 °C for 2 h to obtain crystalline hematite nanorod films followed by a second annealing at 700 °C for 10 min to achieve higher crystallinity.

### 2.2. Characterization

X-ray diffraction (XRD) patterns were collected by using a PW3040/60 X'per PRO, PANalytical, using Cu-Kα (*λ* = 1.54056 Å) radiation, an accelerating voltage of 40 kV, and a current of 30 mA. Raman spectra were obtained using a 0.2 mW 532 nm laser (AFM-Raman, WITec, alpha300R). Ultraviolet-visible absorbance was measured by using a UV-Vis spectrometer (UV-2401 PC, Shimadzu). Surface morphology and structure were investigated using a scanning electron microscope (SEM-S4800, HITACHI) and transmission electron microscope (TEM, JEOL, JEM-2100F, 200 kV). High-angle annular dark-field (HAADF) and energy dispersive X-ray spectroscopy (EDS) analyses were carried out using a FEI Titan3 G2 60-300 microscope equipped with a double-sided Cs corrector. The surface atomic composition and atomic depth profiling were performed by X-ray photoelectron spectroscopy (XPS, Thermo-Fisher, Kα). Time-of-flight secondary ion mass spectrometry (TOF-SIMS) data were obtained on an instrument (ION-TOF GmbH, Germany) using a 10 keV Bi^+^ primary ion beam source.

### 2.3. Photoelectrochemical performance measurements

Solar water oxidation was performed in a three-electrode cell with the photoanode as a working electrode, a platinum mesh as a counter electrode and Ag/AgCl as a reference electrode in NaOH (1 M, pH = 13.6) electrolyte. Photocurrent (*J*)–potential (*V*) curves and electrochemical impedance spectra (EIS) were recorded under simulated solar light generated by a solar simulator (91170, Oriel) with an air mass 1.5 G filter. Light intensity of the solar simulator was calibrated to 1 sun (100 mW cm^−2^) using a reference cell certified by the National Renewable Energy Laboratories (USA). All electrochemical measurements were performed on a potentiostat (IviumStat, Ivium Technologies). The Mott–Schottky plots were measured by sweeping the 0.4–1.0 V_RHE_ range with an alternative current (AC) frequency of 1000 Hz under dark conditions. EIS spectra were recorded at 1.23 V_RHE_ with an AC potential frequency range of 100 000–0.1 Hz. Z-View software (Scribner Associates) was used for fitting the experimental EIS data to an equivalent circuit model. The incident photon-to-electron conversion efficiency (IPCE) was measured using a Xe lamp (300 W, Oriel) and a monochromator with a bandwidth of 10 nm at 1.23 V_RHE_ in the same electrolyte. The on-line gas evolution analysis of H_2_ and O_2_ was conducted using a gas chromatograph (Agilent, GC 7890) equipped with a packed column (Supelco, Carboxen 1000) and a thermal conductivity detector (TCD).

The electrochemically active surface area (EASA) was determined by measuring the capacitive current associated with double-layer charging from the scan-rate dependence of cyclic voltammograms (100–200 mV s^−1^). The double layer capacitance (*C*_dl_) was evaluated by plotting Δ*J* = (*J*_a_ − *J*_c_) at 1.1 V_RHE_ against the scan rate, in which the linear slope is equivalent to twice *C*_dl_. The charge carrier density (*N*_D_) is inversely proportional to the slope and can be extracted using the following equation:
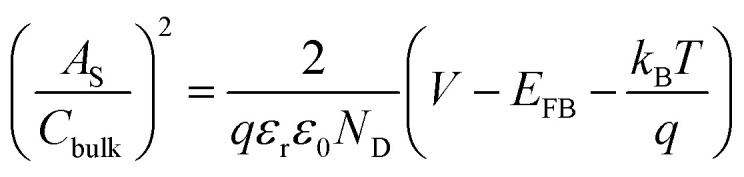
where 
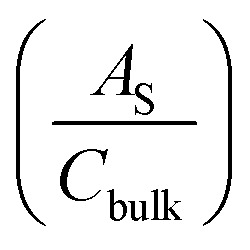
 is the surface area-corrected space charge capacitance, *V* is the applied potential, *E*_FB_ is the flat band potential of the electrode, *ε*_r_ = 32, *ε*_0_ = 8.85 × 10^−12^ C^2^ J^−1^ m^−1^, *q* = 1.602 × 10^−19^ C, *T* = 298 K, and *k*_B_ = 1.38 × 10^−23^ J K^−1^ for hematite.

## 3. Results and discussion

### 3.1. Fabrication of the F,Ti:Fe_2_O_3_ nanorod photoanode

**Fig. 1:**
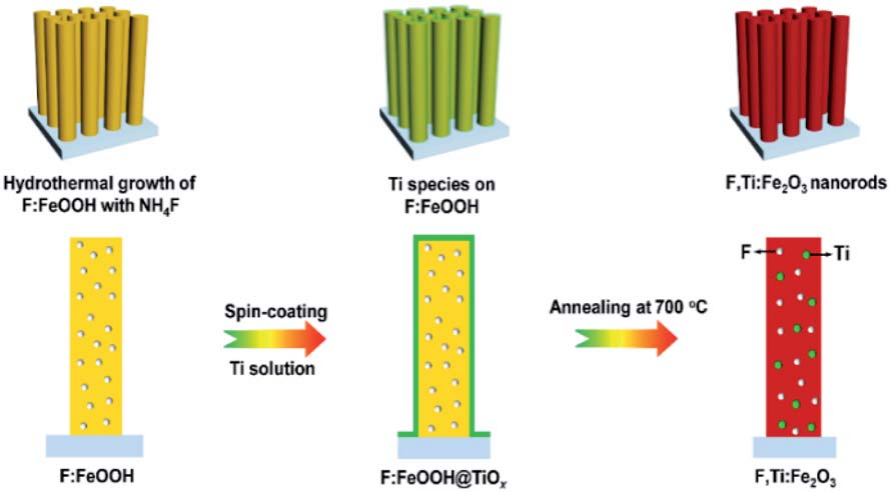
Schematic synthesis procedure of F,Ti:Fe_2_O_3_ nanorods *via* the two-step co-doping process of *in situ* F-doping and *ex situ* external Ti-doping.

A schematic synthesis procedure of F,Ti:Fe_2_O_3_ nanorods grown on FTO glass is shown in Fig. 1. First, F-doped β-FeOOH nanorods on FTO glass were synthesized by a hydrothermal reaction in NH_4_F-containing solution to achieve *in situ* F-doping (Fig. S1 in the ESI†). Then, a dilute titanium butoxide solution was uniformly deposited on the surface of F:FeOOH nanorods by spin-coating at a speed of 2000 rpm for 20 s. Finally, titanium-deposited F:FeOOH nanorods were annealed at 550 °C for 2 h and then 700 °C for 10 min to execute *ex situ* Ti-doping and complete the two-step co-doping process that gave the F,Ti:Fe_2_O_3_/FTO photoanode. Annealing of all the photoanodes was carried out under the air conditions. Moderate conditions (instead of 800 °C or longer time) were selected to compromise between the loss of doped fluorine and the high crystallinity obtained during the transformation of FeOOH into Fe_2_O_3_.^21^ The pristine Fe_2_O_3_, single-doped F:Fe_2_O_3_, and Ti:Fe_2_O_3_ photoanodes were also synthesized according to the same procedure except for the doping steps. Single-step co-doping was also tried for comparison through either all *in situ* or all *ex situ* doping.

### 3.2. Morphology of the F,Ti:Fe_2_O_3_ nanorod photoanode

**Fig. 2:**
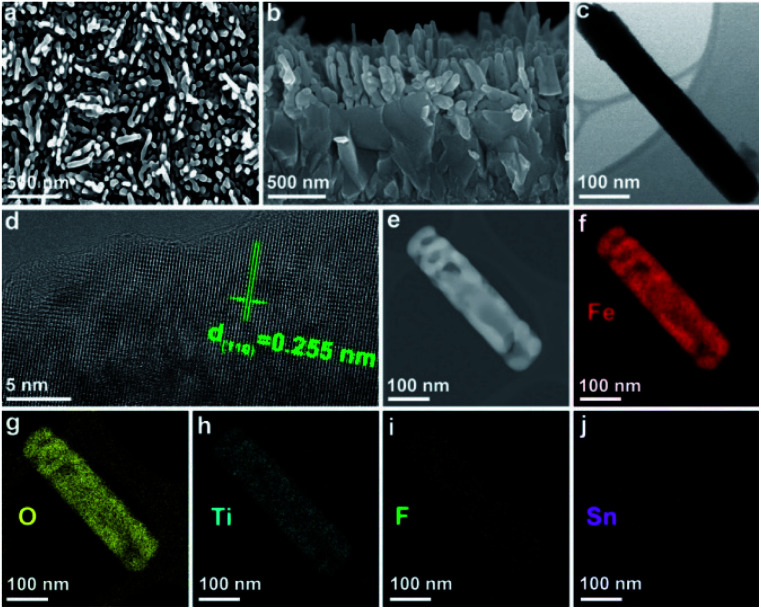
SEM (top view (a) and cross-sectional (b)), TEM (c), HRTEM (d), HAADF (e), and elemental mapping images of Fe (f), O (g), Ti (h), F (i), and Sn (j) of F,Ti:Fe_2_O_3_ nanorods.

SEM images of the F,Ti:Fe_2_O_3_ photoanode in Fig. 2a and b show the morphology of the annealed nanorods with diameters of 80–120 nm and lengths of 350–500 nm. All three photoanodes (F:Fe_2_O_3_, Ti:Fe_2_O_3_, and F,Ti:Fe_2_O_3_) exhibit an identical nanorod morphology, but Ti:Fe_2_O_3_ and F,Ti:Fe_2_O_3_ photoanodes show relatively smaller diameters compared with Fe_2_O_3_ and F:Fe_2_O_3_ (Fig. S2†). This suggests that the excess titanium remaining on the surface suppresses the agglomeration of Fe_2_O_3_ nanorods in spite of the rather high annealing temperature (700 °C).^22^ The TEM image of a single nanorod in Fig. 2c shows a diameter of ∼78 nm and a length of ∼515 nm, and the high-resolution TEM (HRTEM) image in Fig. 2d displays a single crystalline nanorod with a *d*-spacing of 0.255 nm corresponding to the (110) crystal plane of hematite. Some nanorods exhibit a porous structure (Fig. 2e), probably because NH_4_F evolves NH_3_ gas during the hydrothermal synthesis. Elemental mapping images of Fe, O, Ti, and F (Fig. 2f–i) show the spatially uniform distribution, indicating the homogeneity of co-doping *via* the two-step process; *in situ* F anion doping followed by *ex situ* Ti cation doping. There is no significant Sn signal that might have diffused from FTO (Fig. 2j), suggesting that the thermal damage of FTO could be negligible.

### 3.3. Physical characterization of F,Ti:Fe_2_O_3_/FTO photoanodes

**Fig. 3:**
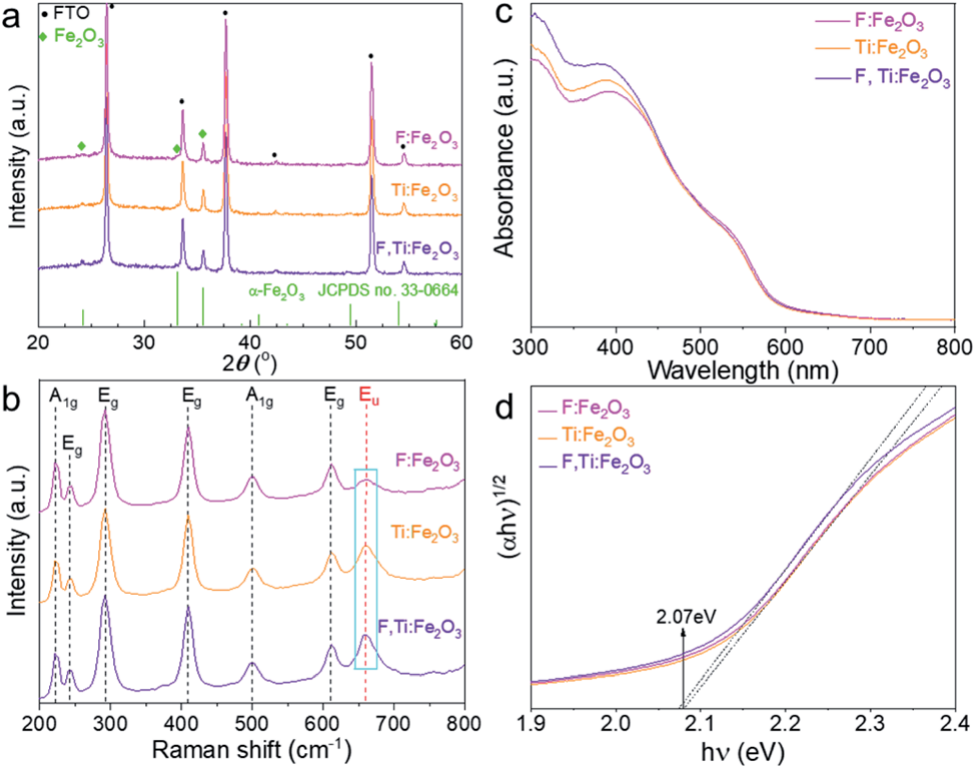
XRD patterns (a), Raman spectra (b), light absorption spectra (c) and Tauc plots (d) of Fe_2_O_3_, F:Fe_2_O_3_, Ti:Fe_2_O_3_, and F,Ti:Fe_2_O_3_ photoanodes.

All of the hematite photoanodes show similar XRD patterns of the rhombohedral α-Fe_2_O_3_ phase (JCPDS no. 33-0664) in Fig. 3a, which demonstrates that doping F and/or Ti does not change the crystal structure of hematite. They all show a strong (110) peak at 2*θ* = 35.61°, indicating a highly orientated growth. Compared with Ti:Fe_2_O_3_, F:Fe_2_O_3_ shows a very weak peak of (104) at 2*θ* = 33.15^°^, indicating that fluorine doping further enhances the orientated crystal growth and gives a higher intensity ratio of (110)/(104) peaks (Fig. S3†). It is known that the electron transport along the hematite (110) plane is four orders-of-magnitude higher than that of the (104) plane,^23^ and such a directional charge flow is quite favourable for high PEC performance. While *in situ* doped fluorine is involved in the crystallization process of hematite, titanium doping does not show this phenomenon since titanium is introduced *ex situ* from the external surface and has no way to exert an influence on the already-constructed crystal structure.

All Raman spectra in Fig. 3b match well with those of hematite with no impurity phase like maghemite (γ-Fe_2_O_3_) or magnetite (Fe_3_O_4_). The peaks at 229 cm^−1^ and 500 cm^−1^ are assigned to the A_1g_ mode, and the other four peaks at 249, 295, 414, and 615 cm^−1^ are assigned to E_g_ modes.^24^ In general, the A_1g_ mode is related to the symmetric stretching of oxygen atoms, while the E_g_ mode is related to symmetric and asymmetric bending of oxygen with respect to iron in the tetrahedral voids. In particular, the forbidden vibration mode (E_u_) at ∼660 cm^−1^ (marked rectangle in Fig. 3b) represents structural disorders.^25,26^ Thus, Ti:Fe_2_O_3_ shows an increase of the E_u_ peak compared with F:Fe_2_O_3_, while F,Ti:Fe_2_O_3_ displays the highest peak intensity, demonstrating that *in situ* F^−^ doping generates little disorder in the structure, whereas *ex situ* Ti^4+^ doping produces much lattice stress or local lattice disorder. The variation of the E_u_ mode caused by F^−^ and/or Ti^4+^ doping demonstrates successful introduction of the dopants into the hematite lattice.

Both Ti,F:Fe_2_O_3_ and Ti:Fe_2_O_3_ show slightly improved light absorption as shown in Fig. 3c compared with F:Fe_2_O_3_. Interestingly, light absorption by F:Fe_2_O_3_ is lower at 420 nm but becomes higher at 550 nm than that by Ti:Fe_2_O_3_, suggesting different mechanisms of absorption enhancement induced by F^−^ or Ti^4+^ doping. This is probably related to their different doping mechanisms. The *in situ* doped fluorine anions prefer to substitute oxygen atoms of Fe_2_O_3_, while the titanium cations substitute iron atoms. Xie *et al.* reported that F^−^ doped hematite generated a defect level to reduce apparent band gap, which led to a significant enhancement of light absorption.^27^ Ti^4+^ doping might induce some intra-band gap states as well, leading to a narrow band gap.^28^ Consequently, F,Ti:Fe_2_O_3_ shows a highest absorption at 420 nm but a lower one at 550 nm than that of F:Fe_2_O_3_. Light harvesting efficiency (LHE, defined as 1–10^−*A*^ where *A* is the measured absorbance) is used to evaluate the light absorption capability of electrodes (Fig. S4†). Compared with single-doped hematite, the co-doped hematite shows slightly higher LHE. Specifically, F:Fe_2_O_3_ shows the highest LHE above the wavelength of 500 nm. From the Tauc plots (Fig. 3d), however, all the derived band gaps are almost the same (∼2.07 eV), indicating that F or Ti doping does not introduce significant intra-band levels in the band gap of hematite, which is in accordance with the literature reports.^29^

### 3.4. The states and compositions of dopants in F,Ti:Fe_2_O_3_

**Fig. 4:**
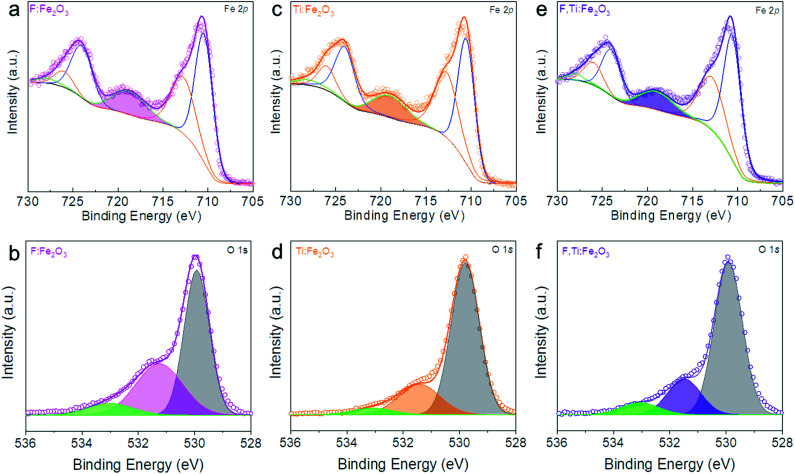
Fe 2p and O 1s XPS spectra of F:Fe_2_O_3_ (a) and (b), Ti:Fe_2_O_3_ (c) and (d) and F,Ti:Fe_2_O_3_ (e) and (f). The coloured peaks in (a), (c), and (e) denote the Fe^3+^ satellite peak. Core-level O 1s XPS spectra in (b), (d), and (f) show the Fe–O bond, oxygen vacancy, and surface hydroxyl group peaks, respectively, from low to high binding energies.

XPS in Fig. 4 shows Fe 2p_1/2_, Fe 2p_3/2_ and Fe^3+^ satellite peaks at 724.5, 711.1 and 718.8 eV, respectively, indicative of α-Fe_2_O_3_.^30^ The F:Fe_2_O_3_ and F,Ti:Fe_2_O_3_ photoanodes show a little larger areas of Fe^3+^ satellite peaks relative to Ti:Fe_2_O_3_, suggesting that the positive charges of Fe sites are enhanced in hematite lattices by the strong electronegativity of F dopants. On the other hand, Ti:Fe_2_O_3_ shows a smaller area of Fe^3+^ satellite peaks relative to that of F:Fe_2_O_3_, indicating that the positive charges of Fe sites were weakened or Fe^2+^ ions were generated in hematite lattices by electronic contribution of Ti dopants. The F dopant in F:FeOOH, F:Fe_2_O_3_ and F,Ti:Fe_2_O_3_ can be verified by the F 1s XPS peak at ∼684.1 eV (Fig. S5†), which is consistent with fluorine in metal fluorides and demonstrates the presence of Fe–F bonds in F-doped hematite.^31^ Both F:Fe_2_O_3_ and F,Ti:Fe_2_O_3_ photoanodes show a lower intensity than that of F:FeOOH, indicating some loss of F dopants during the annealing process. However, F,Ti:Fe_2_O_3_ shows a slightly higher intensity of F dopants than that of F:Fe_2_O_3_, suggesting that *ex situ* Ti doping can suppress the loss of F dopants to some extent. This could be a source of the synergistic promotion effect of F and Ti co-doping. This result was further demonstrated by TOF-SIMS in Fig. S6.† The fluorine signals from F:Fe_2_O_3_ and F,Ti:Fe_2_O_3_ are almost an order-of-magnitude higher than Ti:Fe_2_O_3_, thereby confirming the success of *in situ* F doping. In addition, F,Ti:Fe_2_O_3_ shows slightly stronger fluorine signals relative to that of F:Fe_2_O_3_, which verifies again fluorine preservation by the excess Ti remaining on the external surface of Fe_2_O_3_ nanorods.

The XPS peaks at ∼464 and 458 eV in Fig. S7† are assigned to Ti 2p_1/2_ and Ti 2p_3/2_ of Ti^4+^ states, respectively.^32^ Importantly, the interaction between F and Ti dopants in F,Ti:Fe_2_O_3_ is manifested in Ti 2p_1/2_ and Ti 2p_3/2_ peaks of F,Ti:Fe_2_O_3_, which show a shift of 0.15 eV towards higher binding energies relative to those of Ti:Fe_2_O_3_, indicating that F dopants also promote the positive charges of Ti dopants because of their strong electronegativity. During annealing, the doped fluorine would tend to escape from the hematite lattice, while the external surface-coated titanium would attempt to go into the lattices. Consequently, the already-doped fluorine is retained more in the lattice by the *ex situ* Ti doping from the external surface. The F and Ti atomic concentrations in the F,Ti:Fe_2_O_3_ photoanode determined by TOF-SIMS are 2% and 3%, respectively (Fig. S8†), but 1.5% F and 4.7% Ti are determined on the surface. This more significant concentration gradient of Ti is reasonable considering the doping procedure – *in situ* F-doping followed by *ex situ* external Ti-doping.

In addition, Sn diffusion from FTO is usually inevitable during the high-temperature annealing process,^33^ although the elemental mapping image in Fig. 2j did not show a significant Sn signal. Thus, the Sn signal was monitored by more sensitive TOF-SIMS for F,Ti:Fe_2_O_3_ photoanodes treated at different annealing temperatures (Fig. S9†). The Sn signal appears from the sample annealed at 700 °C, and gets stronger for the samples annealed at 800 and 900 °C. Therefore, F,Ti:Fe_2_O_3_ actually contains a small amount of Sn dopant diffused from FTO in addition to intentional dopants of F and Ti. The lattice oxygen peak of F,Ti:Fe_2_O_3_ shows a little shift (only ∼0.1 eV) towards higher binding energies from that of Fe_2_O_3_, probably due to strong electron-withdrawing capability of F dopants. Deconvoluted O 1s spectra in Fig. 4b, d and f show the Fe–O bond, oxygen vacancy, and surface hydroxyl group peaks, respectively, from low to high binding energies. The F:Fe_2_O_3_ photoanode shows the largest area of oxygen vacancies (middle peak) due to the substitution of oxygen and surface trap states, while Ti:Fe_2_O_3_ shows a relatively small area due to the passivation of surface trap states by *ex situ* Ti doping. Importantly, the co-doped F,Ti:Fe_2_O_3_ photoanode contains an intermediate amount of oxygen vacancies. An appropriate amount of oxygen vacancies could significantly contribute to the enhanced PEC performance by improving the electrical conductivity of the photoanode.^11,34^

### 3.5. Photoelectrochemical water oxidation performance

The PEC water oxidation performance of the optimized F,Ti:Fe_2_O_3_ photoanode was studied under simulated 1 sun irradiation (100 mW cm^−2^) in 1 M NaOH electrolyte in a three-electrode cell with the photoanode, Ag/AgCl (3 M NaCl), and Pt mesh as the working, reference, and counter electrodes, respectively. The F:Fe_2_O_3_ photoanode with the optimized F dopants (by addition of 30 mg NH_4_F in the precursor solution, Fig. S10†) shows a photocurrent density of 0.35 mA cm^−2^ at 1.23 V_RHE_. The F:Fe_2_O_3_ photoanode with optimized Ti^4+^ dopants (by spin-coating of 10 mM TiCl_4_ solution, Fig. S11†) generates a photocurrent of 0.90 mA cm^−2^ at 1.23 V_RHE_, which is ∼3 times that of F:Fe_2_O_3_.

**Fig. 5:**
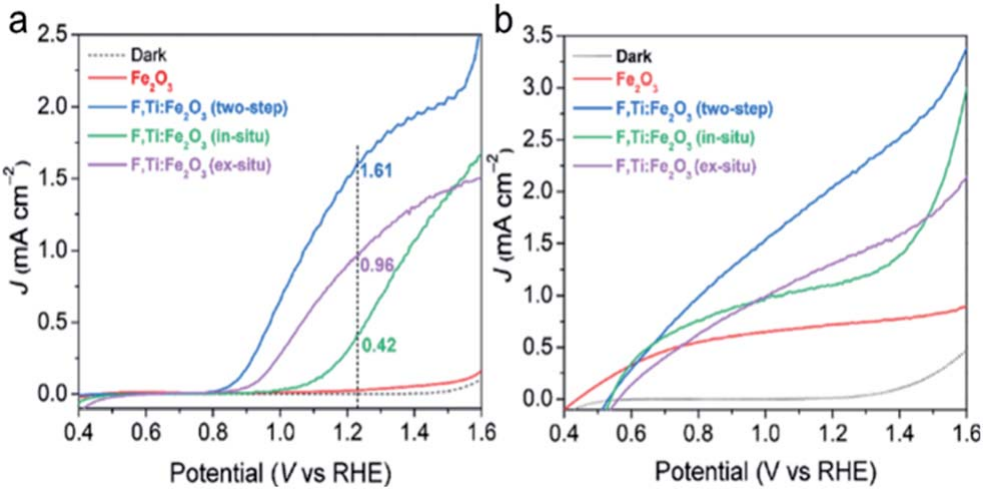
Comparison of single-step (*in situ* or *ex situ*) and two-step (*in situ* followed by *ex situ*) co-doping of F and Ti into hematite to fabricate the F,Ti:Fe_2_O_3_ photoanode. (a) *J*–*V* curves of water oxidation (without H_2_O_2_). (b) *J*–*V* curves of H_2_O_2_ oxidation. The PEC water oxidation was performed under 1 sun irradiation (100 mW cm^−2^) in 1 M NaOH electrolyte.

In the study of the performance of PEC water oxidation over these modified hematite photoanodes, first of all, we would like to verify the efficacy of our two-step co-doping strategy relative to a single step co-doping that is either *in situ* only or *ex situ* only. As shown in the photocurrent density (*J*)–applied voltage (*V*) curves in Fig. 5a, the photocurrent generation at 1.23 V_RHE_ under 1 sun irradiation over the F,Ti:Fe_2_O_3_ photoanode co-doped by the two-step process is much higher (1.6 mA cm^−2^ at 1.23 V_RHE_) than that over the photoanode co-doped in an *ex situ* single step (0.96 mA cm^−2^) or *in situ* single step (0.42 mA cm^−2^). The *J*–*V* curves in Fig. 5b represent the performance of the photoanodes for photo-oxidation of a hole scavenger (0.5 M H_2_O_2_) in the same electrolyte. Since the surface charge recombination of the highly reactive H_2_O_2_ is negligible, this performance represents charge transfer characteristics in the bulk of the photoanode. In this case as well, the F,Ti:Fe_2_O_3_ photoanode shows a similar trend of the performance gaps depending on the co-doping method. Thus, our two-step co-doping strategy produces the F,Ti:Fe_2_O_3_ photoanode that demonstrates the synergy effect of co-doping in the most prominent manner. As mentioned, the second step of external Ti doping can suppress the drain of already-doped F and agglomeration of hematite nanorods during the high temperature annealing process. These beneficial side effects cannot be expected for *in situ* or *ex situ* single-step doping methods.

**Fig. 6:**
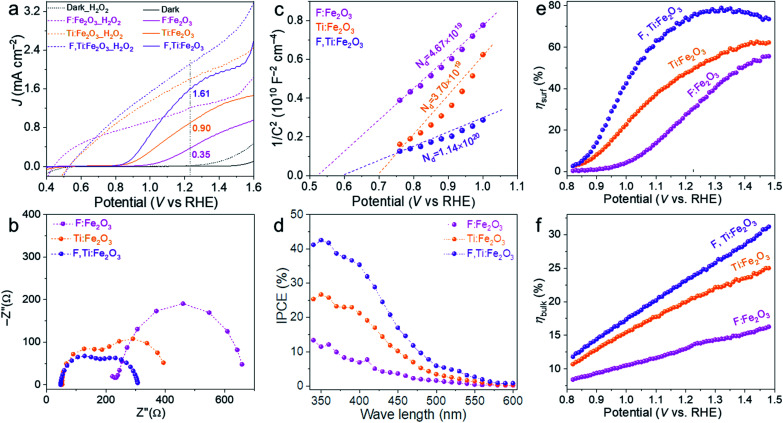
*J*–*V* curves with (dashed) and without (solid) the H_2_O_2_ hole scavenger in electrolyte (a), Nyquist plots (b), Mott–Schottky plots (c), IPCE (d), *η*_surf_ (e) and *η*_bulk_ (f) of F:Fe_2_O_3_, Ti:Fe_2_O_3_, and F,Ti:Fe_2_O_3_. The PEC water oxidation was performed under 1 sun irradiation (100 mW cm^−2^) in 1 M NaOH electrolyte.

The *J*–*V* curves of different photoanodes with (dotted lines) and without H_2_O_2_ (solid lines) are summarized in Fig. 6a. Compared with single F- or Ti-doped Fe_2_O_3_ photoanodes, F and Ti co-doped hematite shows synergistically enhanced PEC performance for oxidation of water as well as H_2_O_2_. The photocurrent onset potential (*V*_on_) is another important kinetic parameter, which is usually related to Fermi-level pinning due to surface trap states. It can be determined from the first-order derivative of the *J*–*V* curve (Fig. S12†).^35^

The result shows that F and Ti dopants exert significant influences on *V*_on_. F-Doping (F:Fe_2_O_3_) causes a negative shift of *V*_on_ by 210 mV induced by its highly polarized surface and facile surface reaction, while Ti-doping (Ti:Fe_2_O_3_) shifts *V*_on_ by 340 mV towards the negative direction by the passivation of surface trapping states. Note that F-doping cannot eliminate most of the surface trapping states while Ti-doping can passivate them more effectively. The F,Ti:Fe_2_O_3_ photoanode shows a cathodic shift of nearly 400 mV from that of bare Fe_2_O_3_, recording the lowest *V*_on_ of 0.74 V_RHE_, which demonstrates the synergetic effect of F- and Ti-doping. Thus, F-doping promotes a facile surface reaction by surface polarization and increases charge carrier density as a typical impurity doping, which leads to a negative shift of *V*_on_ and improved electrical conductivity. The *ex situ* external Ti-doping from spin-coated titanium also improves the electrical conductivity, but in addition, some Ti remaining on the external surface of Fe_2_O_3_ forms a surface passivation layer that passivates the surface trap states to cause a cathodic shift of *V*_on_.^36^ Indeed, TiO_2_ is the most frequently used material as a passivation layer for many photoanode materials. This inadvertent formation of the Ti passivation layer also provides an additional advantage of the two-step co-doping strategy using *ex situ* Ti doping.

The gases evolved from the F,Ti:Fe_2_O_3_ photoanode and the Pt counter electrode were quantified by gas chromatography (Fig. S13†), which was carried out at a constant potential of 1.30 V_RHE_ for 60 min. The O_2_ and H_2_ gases evolved with their ratio close to the stoichiometry (O_2_/H_2_ = 1/2) as shown in Fig. S14.† The faradaic efficiencies (ratio of gas evolution/photocurrent generation) of O_2_ and H_2_ evolution reactions are 93.5% and 98.5%, respectively (Fig. S15†). The results demonstrate that the measured photocurrents do come mostly from O_2_ and H_2_ evolution reactions during the PEC water splitting without any significant parasitic process.

Another informative indicator of catalytic activity is the EASA, which can be determined by measuring the capacitive current associated with double-layer charging from the scan-rate dependence of cyclic voltammograms (Fig. S16†). Apparently, the Ti-doped photoanode shows a larger EASA than that of the F-doped one, indicating that the Ti dopants not only passivate surface trapping states but also provide more catalytically active sites by suppressing agglomeration of hematite nanorods during the annealing step (Fig. 2 and S2†). Consequently, the F and Ti co-doped photoanode shows more than 2 times higher EASA than that of bare Fe_2_O_3_.

The charge transfer characteristics were investigated by EIS analysis at 1.23 V_RHE_ under 1 sun irradiation (Fig. S17†). The Nyquist plots in Fig. 6b were fitted to a typical two-*RC*-unit equivalent circuit, where *R*_s_ is the sheet resistance involving the electrolyte, FTO resistance and external contact; *R*_trap_/*C*_bulk_ denotes the electron pathway within the bulk of the electrode (the semicircle at the high frequency region); *R*_ct_/*C*_ss_ represents the interface between the electrode and electrolyte (the semicircle at the low frequency region).^37^ Overall, both semicircles at high and low frequency regions gradually become smaller along with the order of F:Fe_2_O_3_ > Ti:Fe_2_O_3_ > F,Ti:Fe_2_O_3_, indicating the decrease of the charge transfer resistance both in bulk and at the interface, which is consistent with the corresponding *J*–*V* curves. Compared with F:Fe_2_O_3_, *R*_s_ values of Ti:Fe_2_O_3_ and F,Ti:Fe_2_O_3_ decrease by 70% and 84%, respectively. In the case of *R*_trap_, F,Ti:Fe_2_O_3_ shows 76% decrease, while *R*_trap_ of Ti:Fe_2_O_3_ decreases by 51% relative to that of F:Fe_2_O_3_. Moreover, *R*_ct_ values of Ti:Fe_2_O_3_ and F,Ti:Fe_2_O_3_ decrease by 50% and 72% relative to that of F:Fe_2_O_3_, respectively. These results indicate that the Ti dopants could make more contribution to the PEC performance of the hematite photoanode than the F dopants. The greatest decrease of all resistances for F,Ti:Fe_2_O_3_ verifies the effectiveness of the synergetic co-doping effect. It should be noted that the concentration of Ti dopants is over 3 times higher than that of F dopants according to TOF-SIMS (Fig. S8†), which indicates that F dopants on the surface promote charge transfer very efficiently. This might arise from the highly polarized surface induced by its strong electronegativity and the facile surface reaction with a low oxygen-evolving overpotential.^24^ In all cases, F and Ti co-doped F,Ti:Fe_2_O_3_ shows a significant synergy effect, resulting in much smaller values of *R*_s_, *R*_trap_ and *R*_ct_.

Mott–Schottky plots (Fig. 6c) were obtained from the bulk capacitance data of EIS spectra, which give the flat band potential (*E*_FB_) from the *x*-intercept and the donor density (*N*_D_) from the slope. All samples have positive slopes, indicating that they are n-type semiconductors.^38^ Compared with Ti:Fe_2_O_3_, F:Fe_2_O_3_ shows 26% increase in *N*_D_ and a negative shift of *E*_FB_ from 0.70 to 0.53 V_RHE_. Although F-doping gives a larger *N*_D_, the increased overpotential (difference between *V*_on_ and *E*_FB_) by the negative shift of *E*_FB_ leads to the sluggish kinetics of water oxidation. The overpotential would cause hole accumulation at the surface and subsequent surface recombination until sufficiently positive potentials are applied for appreciable charge transfer across the interface.^39^ However, Ti doping lowers the overpotential by the positive shift of *E*_FB_ even without a comparable increase of *N*_D_, leading to significantly improved photocurrents. In particular, F,Ti:Fe_2_O_3_ displays a synergistic effect of the two dopants showing the largest amount of *N*_D_ (over 3 times that of Ti:Fe_2_O_3_) and substantially improving the poor electrical conductivity of hematite.

The IPCE represents a quantitative measure of the photoactivity; IPCE = (1240 × *J*_light_)/(*λ* × *P*_light_), where *P*_light_ is the measured irradiance at a specific wavelength (*λ*) of incident light and *J*_light_ is the measured photocurrent density. Single doping (F or Ti) and co-doping improve the IPCE in the entire wavelength range of 340–600 nm (Fig. 6d), showing the trend of F,Ti:Fe_2_O_3_ > Ti:Fe_2_O_3_ > F:Fe_2_O_3_ which is consistent with their corresponding *J*–*V* curves and EIS results. Besides, the independent IPCE can be integrated with the standard AM 1.5G solar spectrum to calculate the photocurrent density using the following equation:
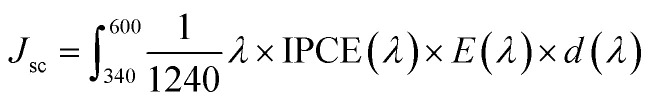
where *J*_sc_ is the integrated photocurrent density, *E*(*λ*) is the solar irradiance at a specific wavelength (*λ*), and IPCE(*λ*) is the photoresponse profile at a specific wavelength (*λ*) at 1.23 V_RHE_. As shown in Fig. S19,† the obtained *J*_sc_ for each photoanode is very close to the experimental values, indicating that *J*_sc_ and IPCE were measured correctly.

The bulk (*η*_bulk_) and surface (*η*_surf_) charge separation efficiencies were obtained by comparing oxidation photocurrents of water and a hole scavenger (0.5 M H_2_O_2_) in Fig. 6a according to the procedure described in Fig. S18.† The *η*_bulk_ represents the fraction of holes that reach the electrode|electrolyte interface without recombination in the bulk, while *η*_surf_ is the fraction of those holes at the interface that is injected successfully into the electrolyte to oxidize water.^40^ As shown in Fig. 6e, *η*_surf_ of F:Fe_2_O_3_ and Ti:Fe_2_O_3_ reaches the maximum of 55% and 62% at 1.45 V_RHE,_ respectively, indicating that F or Ti doping has comparable contribution to *η*_surf_, while F,Ti:Fe_2_O_3_ achieves a higher *η*_surf_ of 78% at a lower potential of 1.3 V_RHE_, indicating a prominent synergy effect of co-doping. In case of *η*_bulk_ (Fig. 6f), Ti-doping (Ti:Fe_2_O_3_ and F,Ti:Fe_2_O_3_) is much more effective than F-doping (F:Fe_2_O_3_). The results of both *η*_surf_ and *η*_bulk_ are consistent with the EIS results.

## 4. Conclusions

For the first time in this work, we studied substitutional co-doping of both Fe^3+^ cations and O^2−^ anions of hematite by Ti^4+^ and F^−^, respectively, to improve the performance of hematite photoanodes for PEC water splitting. In particular, we developed a novel two-step co-doping strategy of *in situ* F^−^ anion doping, followed by *ex situ* Ti^4+^ cation doping. The promotional effects were much more pronounced when our unique two-step doping strategy was employed – *in situ* F-doping followed by *ex situ* external Ti-doping, rather than single-step co-doping strategies that were all *in situ* or all *ex situ*. The *in situ* F-doping induced a large ratio of (110)/(104) crystal planes and a highly polarized surface. *Ex situ* Ti-doping plays multiple roles – improving the electrical conductivity of hematite, passivating surface trapping states, suppressing the agglomeration of hematite nanorods, and protecting already-doped fluorine. As a result, the optimized F,Ti:Fe_2_O_3_ photoanode without any cocatalyst generates a photocurrent density of 1.61 mA cm^−2^ at 1.23 V_RHE_ under 1 sun irradiation. Besides, its *V*_on_ exhibited a cathodic shift of ∼200 mV relative to F:Fe_2_O_3_. This two-step co-doping strategy of *in situ* anions and *ex situ* cations could be applied to design efficient photoelectrodes in general for solar energy conversion.

## Author contributions

Kyoungwoong Kang: methodology, synthesis and characterization of materials, formal analysis, writing original draft. Hemin Zhang: conceptualization, resources, data analysis, writing & editing. Jeong Hun Kim: data discussion, validation. Woo Jin Byun: gas evolution measurements. Jae Sung Lee: supervision, conceptualization, review & editing, project administration.

## Conflicts of interest

There are no conflicts to declare.

## Supplementary Material

NA-004-D2NA00029F-s001
