# Supplementary material for: An in situ fluorine and ex situ titanium two-step co-doping strategy for efficient solar water splitting by hematite photoanodes
Source: Nanoscale Adv. 2022 Feb 12;4(6):1659–67. doi: 10.1039/d2na00029f (PMC9418710; doi:10.1039/d2na00029f)
Supplement: NA-004-D2NA00029F-s001 [file NA-004-D2NA00029F-s001.pdf]

Electronic Supplementary Material (ESI) for Nanoscale Advances.
This journal is © The Royal Society of Chemistry 2022

Electronic Supplementary Information

In-Situ Fluorine and *Ex-Situ* Titanium Two-Step Co-doping Strategy for Efficient Solar Water Splitting by Hematite Photoanodes

Kyoungwoong Kang[a, 1], Hemin Zhang[b, 1]*, Jeong Hun Kim[a], Woo Jin Byun[a], and Jae Sung Lee[a]*

[a]School of Energy and Chemical Engineering, Ulsan National Institute of Science and Technology (UNIST), 50 UNIST-gil, Ulsan 44919 (Republic of Korea)

[b]College of Materials Science and Engineering, Sichuan University, Chengdu 610065, China

[1]These authors contributed equally.

***Corresponding authors**

E-mail addresses: hmzhang@scu.edu.cn (H. Zhang), jlee1234@unist.ac.kr (J. S. Lee).

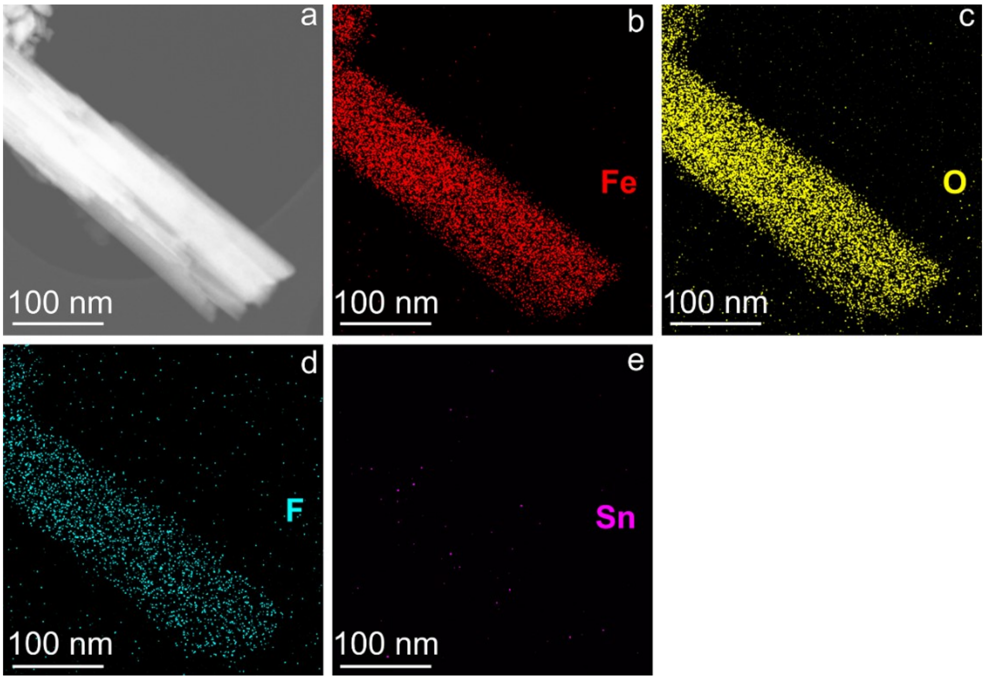

Fig. S1. HAAD (a) and elemental mapping (Fe (b), O (c), F (d), Sn (e)) images of the synthesized F:FeOOH nanorods.

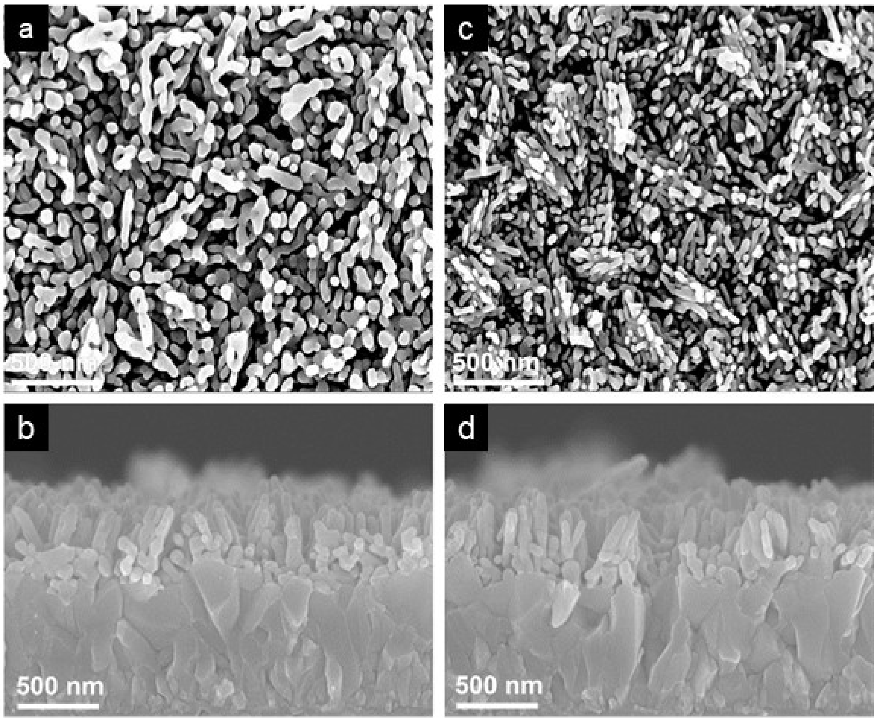

Fig. S2. Top view and cross-section SEM images of F:Fe_2O_3 (**a** and **b**) and Ti:Fe_2O_3 (c and **d**), respectively. Compared with F:Fe_2O_3 samples, Ti:Fe_2O_3 sample shows a little smaller diameter of nanorods, indicating that titanium deposited on the surface of FeOOH nanorods can prevent the diameter of nanorods from becoming bigger during annealing process. Additional benefit of F,Ti:Fe_2O_3 sample is to suppress the drain of the doped fluorine.

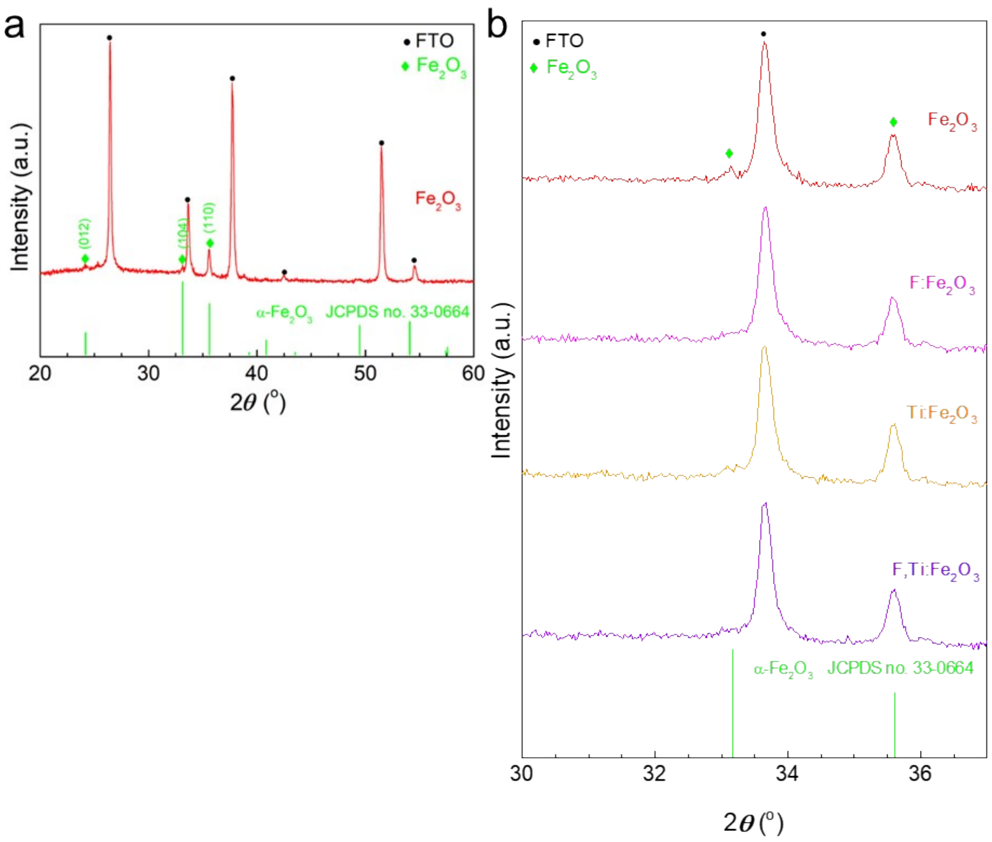

Fig. S3. XRD of pure bare hematite shows an obvious crystal plane of (104) (**a**) and the magnified XRD patterns of all samples correspondingly (**b**).

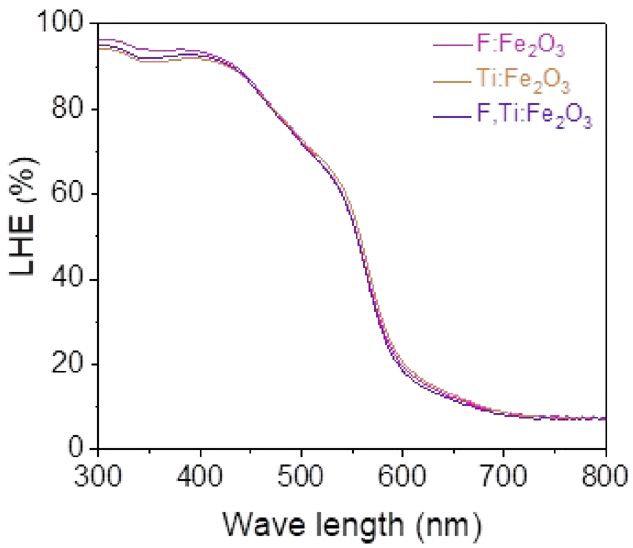

Fig. S4. LHE of F:Fe_2O_3, Ti:Fe_2O_3 and F,Ti:Fe_2O_3.

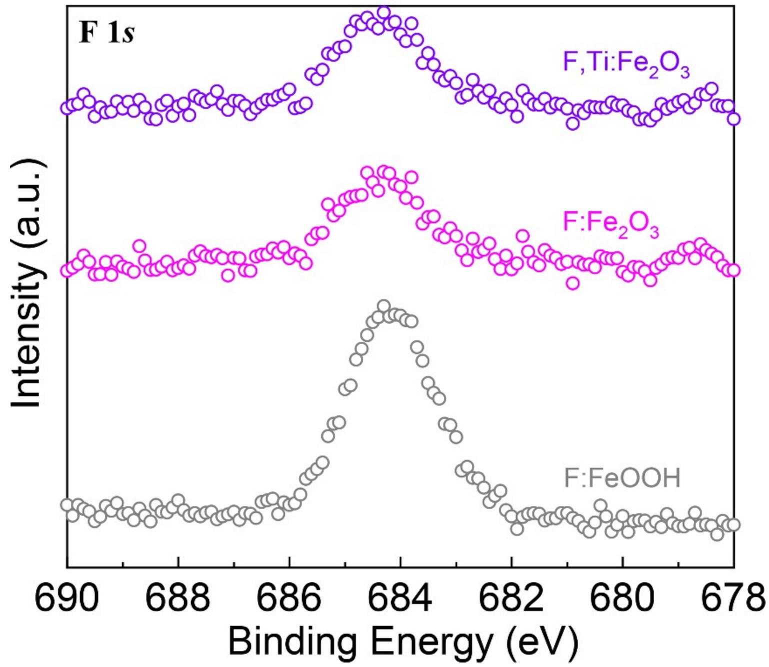

Fig. S5. F 1*s* XPS spectra of F:FeOOH, F:Fe_2O_3 and F,Ti:Fe_2O_3.

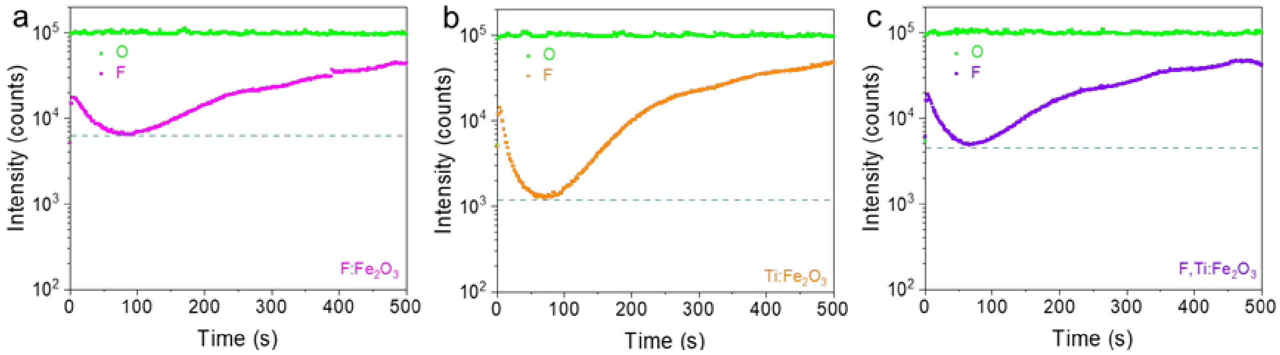

Fig. S6. TOF-SIMS of F:Fe_2O_3 (**a**), Ti:Fe_2O_3 (**b**) and F,Ti:Fe_2O_3 (**c**).

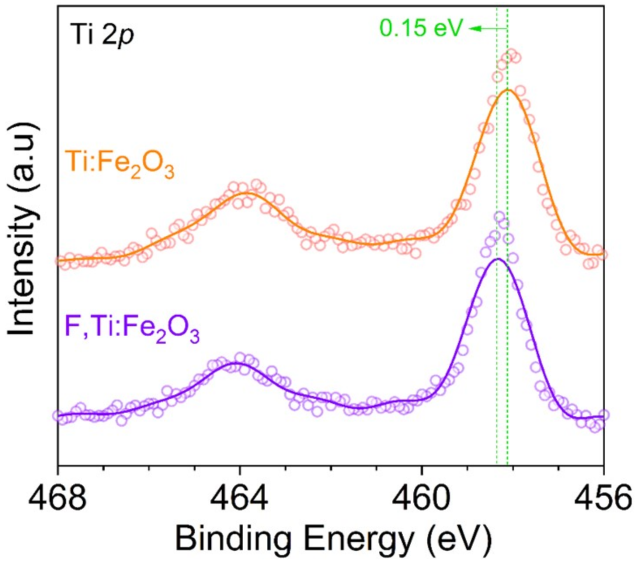

Fig. S7. Ti 2*p* XPS spectra of $Ti:Fe_2O_3$ and $F,Ti:Fe_2O_3$.

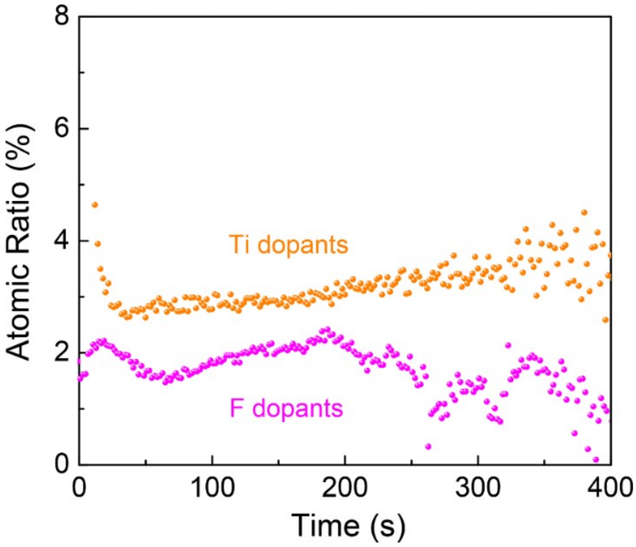

Fig. S8. F and Ti dopants monitored by TOF-SIMS.

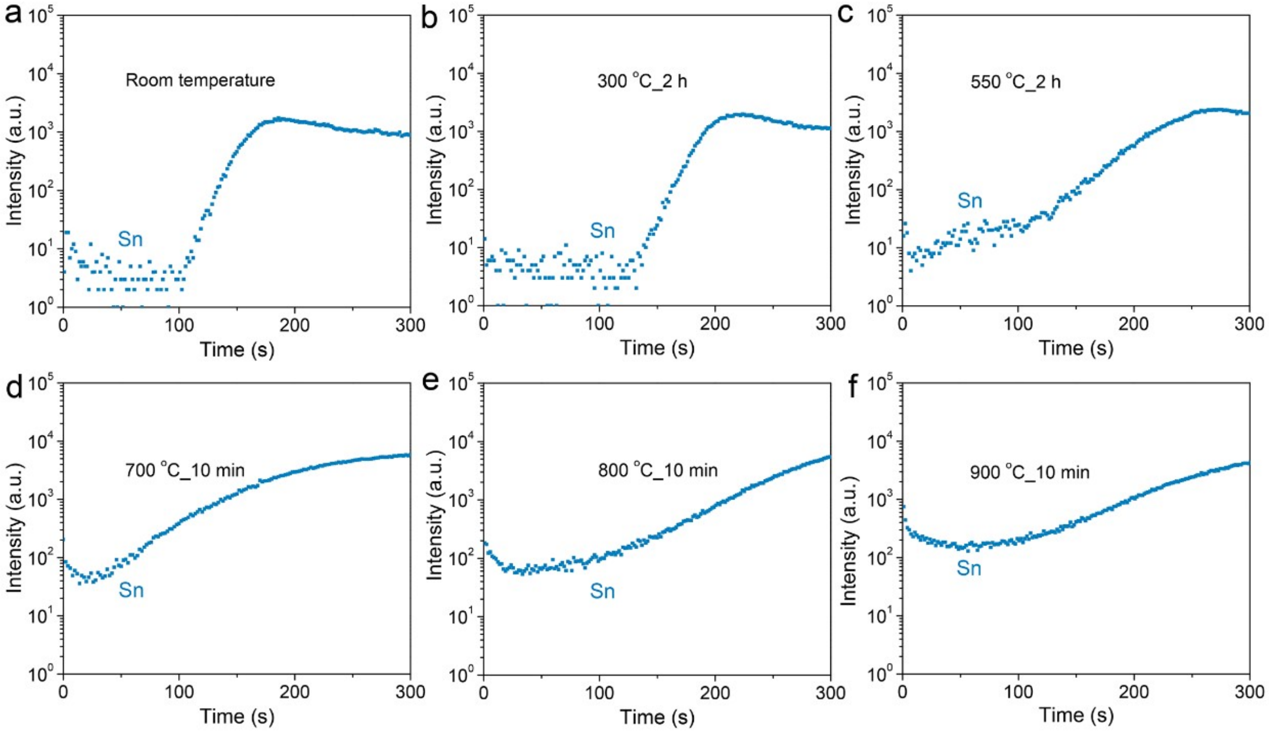

Fig. S9. Sn signal monitored by TOF-SIMS at different annealing conditions. (**a**) Room termperature. (**b**) 300 °C for 2 h. (**c**) 550 °C for 2 h. (**d**) 700 °C for 10 min. (**e**) 800 °C for 10 min. (**f**) 900 °C for 10 min.

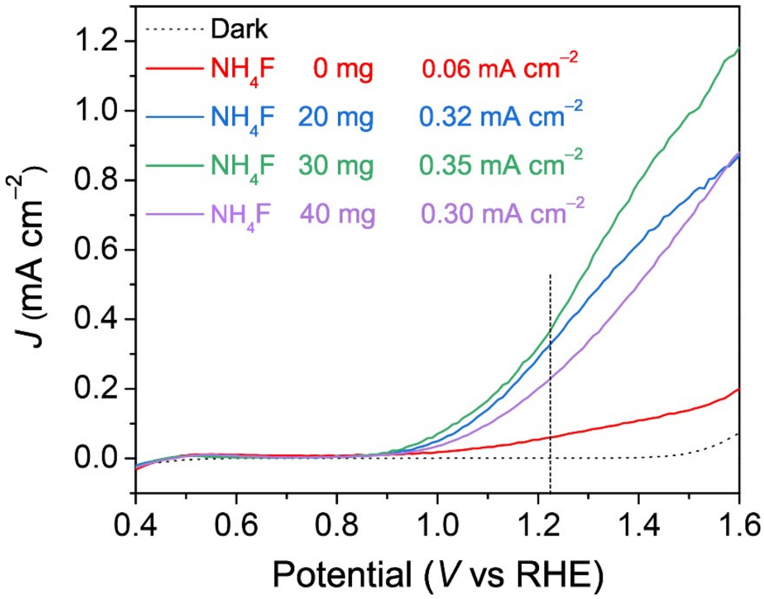

Fig. S10. Optimization of F dopants by addition of different amounts of NH_4F.

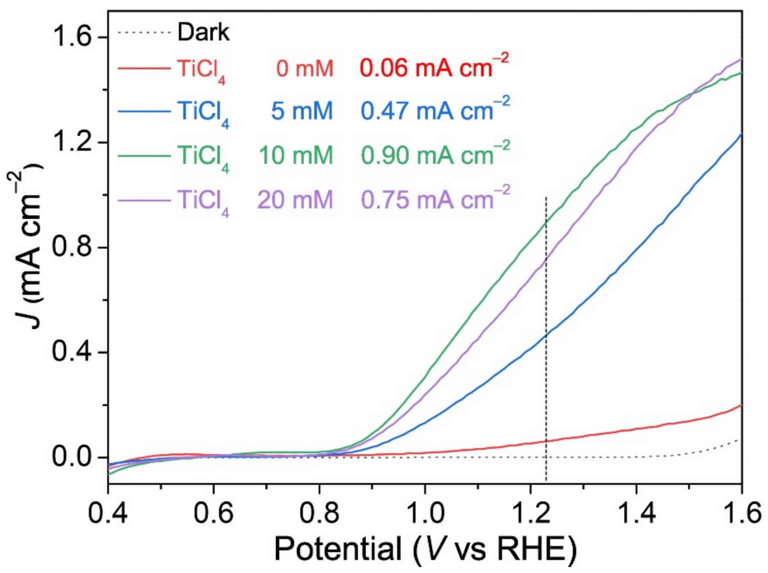

Fig. S11. Optimization of Ti dopants by spin coating of different $TiCl_4$ concentrations.

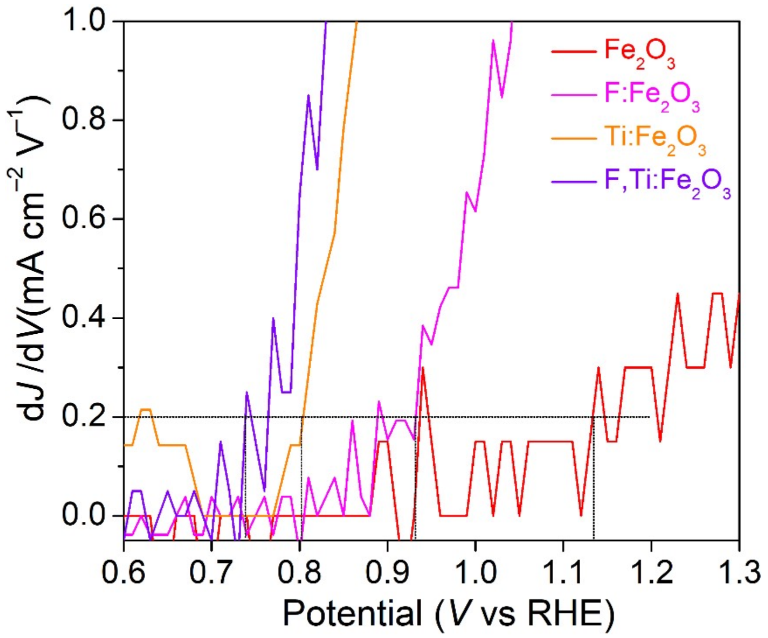

Fig. S12. Onset potential (V_{on}) evaluation by first-order derivative of the photocurrent density as a function of potential. The V_{on} is defined as the value at which dJ/dV > 0.2 mA $cm^{-2}V^{-1}$ from obtained J-V curves. The V_{on} values of bare Fe_2O_3, F:Fe_2O_3, Ti:Fe_2O_3 and F,Ti:Fe_2O_3 are 1.14, 0.93, 0.80 and 0.74 V_{RHE}, respectively.

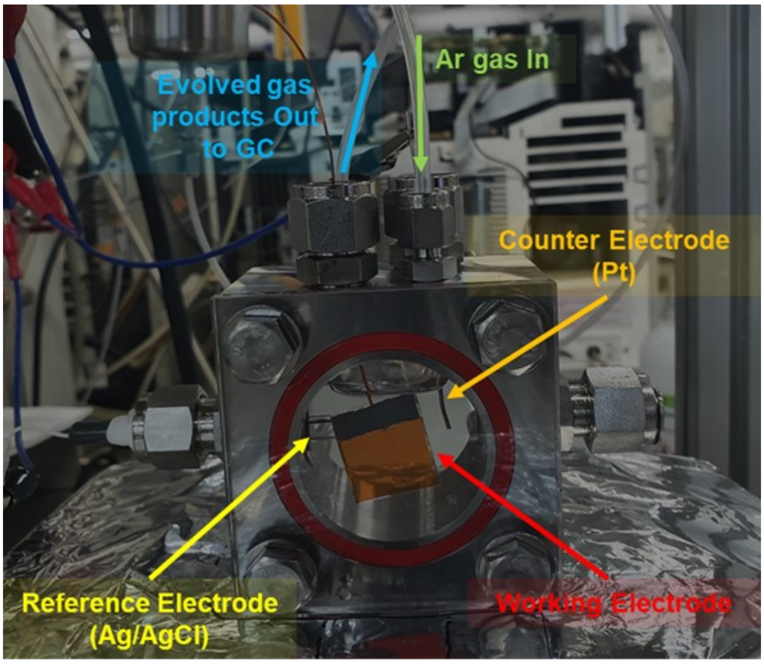

Fig. S13. Digital photograph of the stainless PEC reactor with a closed circulation for the measurement of O_2 and H_2 evolution.

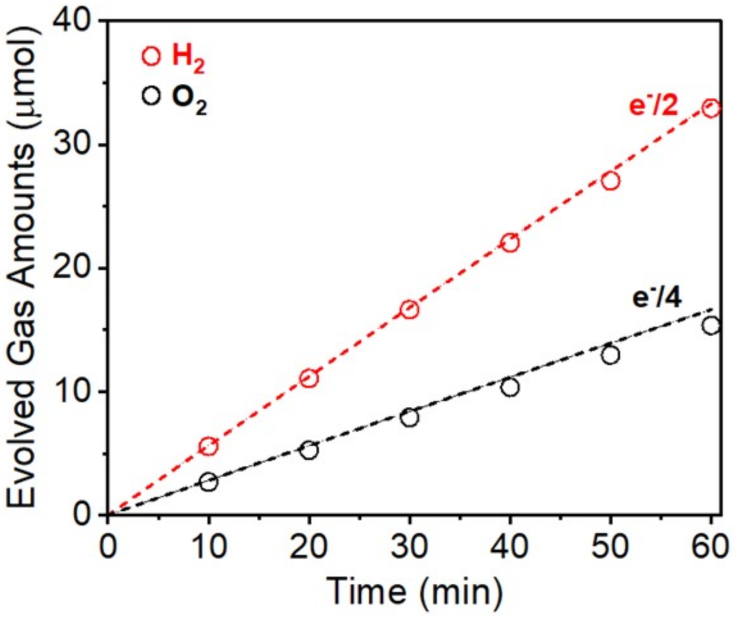

Fig. S14. Gas chromatography of O_2 and H_2 evolved from the F,Ti:Fe_2O_3 photoanode at a constant potential of 1.30 V_{RHE}. The result demonstrates that the ratio of evolved O_2 and H_2 is close to the stoichiometry of H_2O.

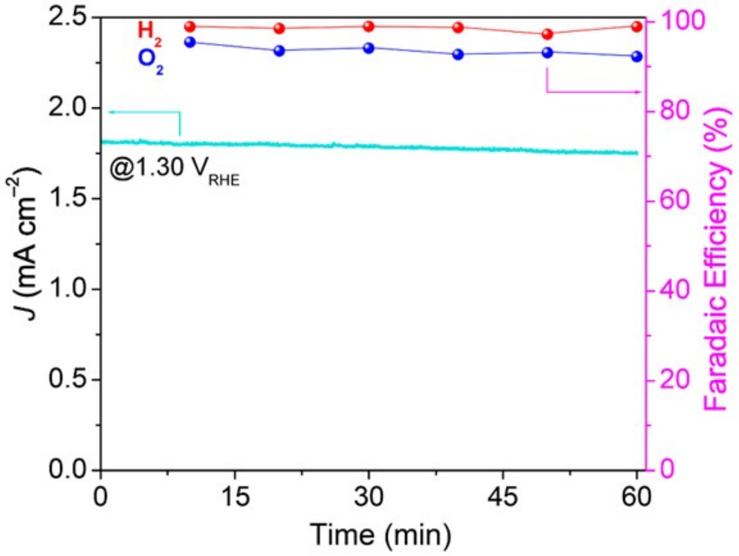

Fig. S15. The stability and Faradaic efficiency (F.E.) of F,Ti:Fe_2O_3 photoanode.
The F.E. can be calculated as follows:

$$\text{F.E.}(\%) = \frac{\textit{moles of the product during the duration}}{\textit{theoretical moles based on measured current}} \times 100\%$$

The F.E. of O_2 and H_2 evolution reaction is 93.5% and 98.5%, respectively.

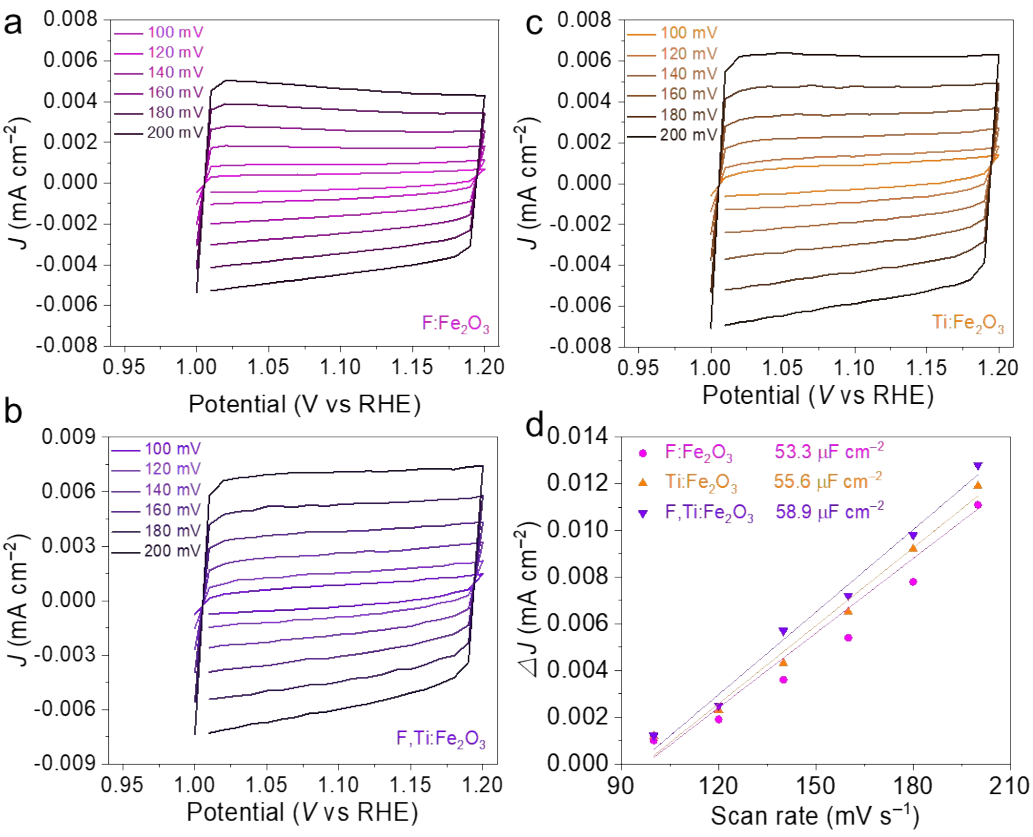

Fig. S16. The fitting of Nyquist plots for Fe_2O_3 (**a**), F:Fe_2O_3 (**b**), Ti:Fe_2O_3 (**c**) and F,Ti:Fe_2O_3 (**d**), respectively. A representative two-RC-unit equivalent circuit (**e**) and the table of corresponding fitting results (f).

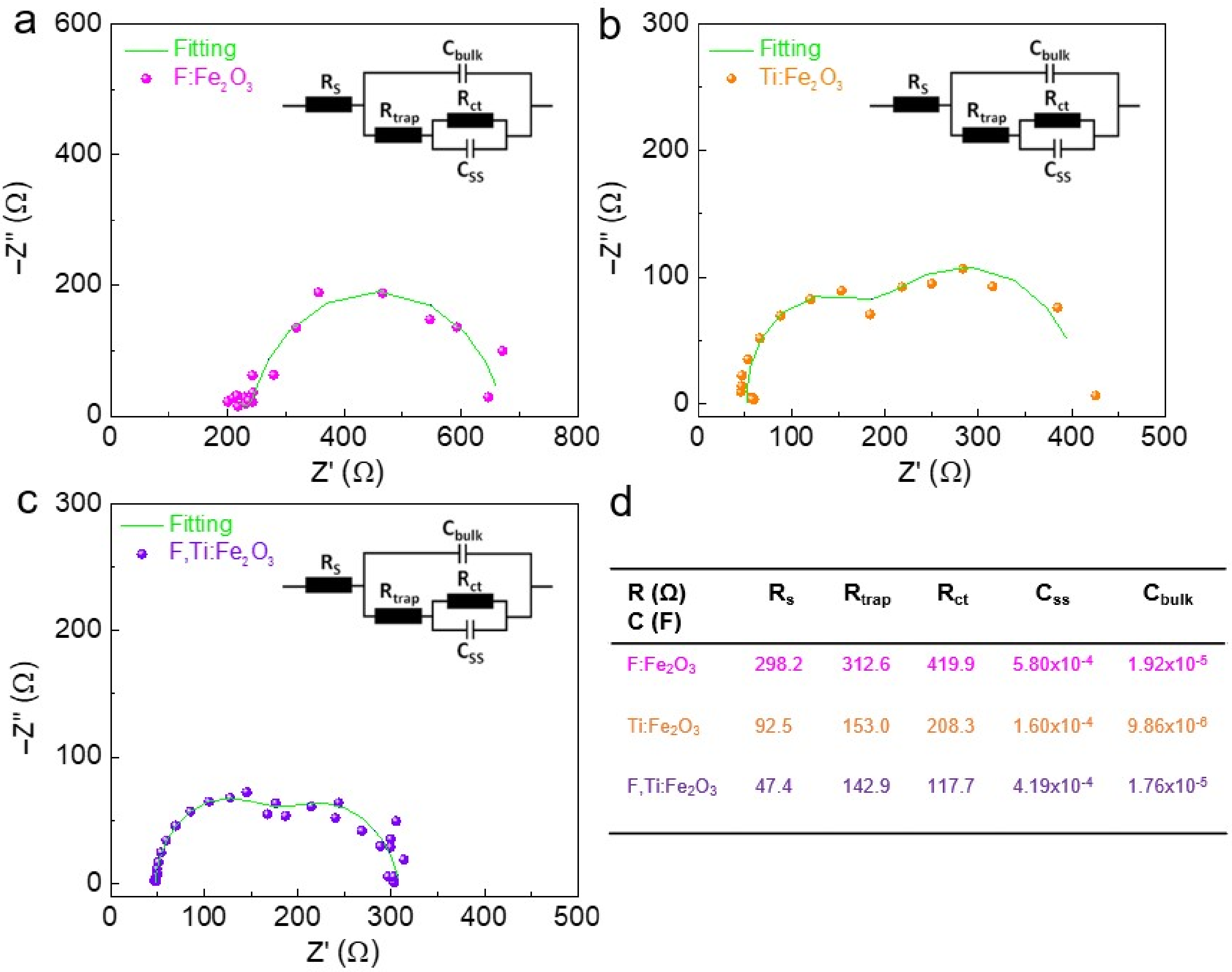

R (Ω) C (F)	R_s	R_{trap}	R_{ct}	C_{ss}	C_{bulk}
F:Fe_2O_3	298.2	312.6	419.9	5.80×10^{-4}	1.92×10^{-5}
Ti:Fe_2O_3	92.5	153.0	208.3	1.60×10^{-4}	9.86×10^{-6}
F,Ti:Fe_2O_3	47.4	142.9	117.7	4.19×10^{-4}	1.76×10^{-5}

Fig. S17. The fitting of Nyquist plots for F:Fe_2O_3 (**a**), Ti:Fe_2O_3 (**b**) and F,Ti:Fe_2O_3 (**c**), respectively. The table of corresponding fitting results (**d**).

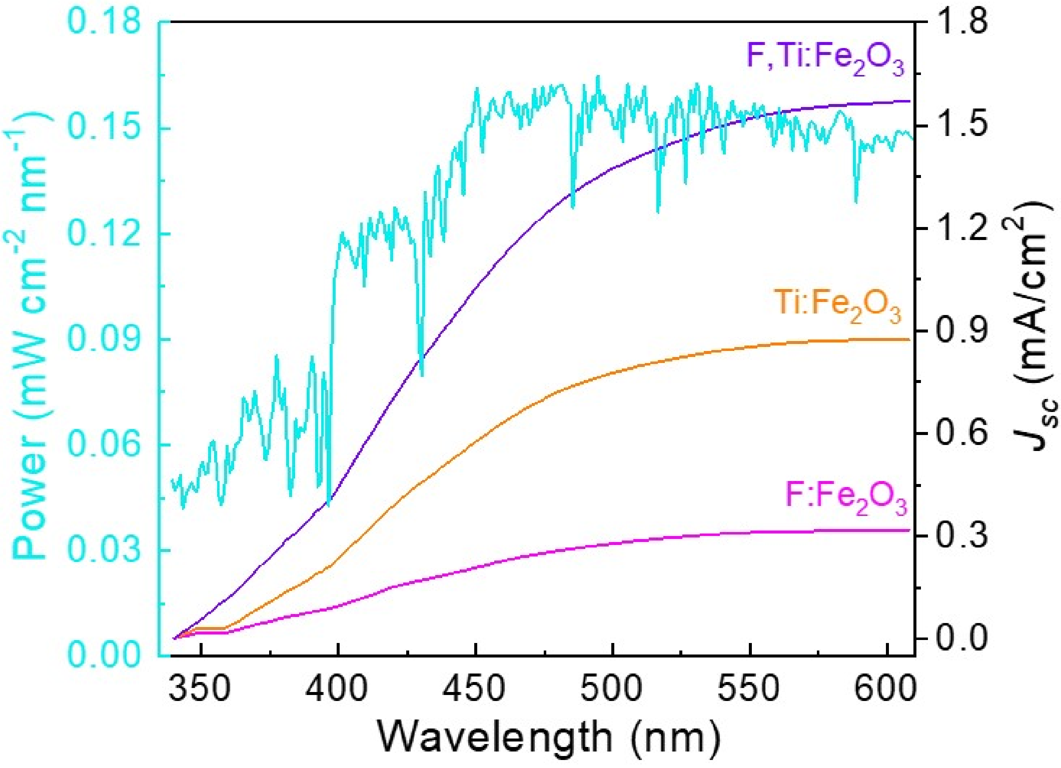

Fig. S18. IPCE integration of F:Fe_2O_3 (0.32 mA cm^{-2}), Ti:Fe_2O_3 (0.87 mA cm^{-2}) and F,Ti:Fe_2O_3 (1.57 mA cm^{-2}) with a standard AM 1.5G solar spectrum (ASTM G-173-03), respectively.

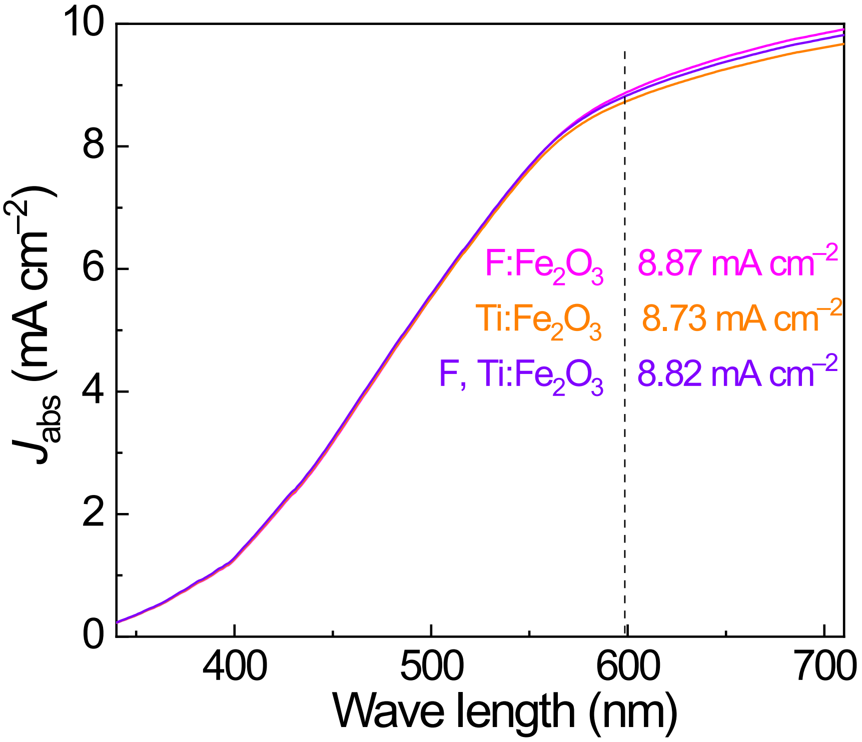

Fig. S19. Absorption photocurrents of J_{abs} for F:Fe_2O_3 (8.87 mA cm^{-2}), Ti:Fe_2O_3 (8.73 mA cm^{-2}) and F,Ti:Fe_2O_3 (8.82 mA cm^{-2}), respectively.

The obtained photocurrent density of J_{H_2O} could be described as following:

$$J_{H_2O} = J_{abs} \times \eta_{bulk} \times \eta_{surface} \quad (1)$$

Since no injection barrier of holes exists for H_2O_2 oxidation ($\eta_{surface} = 1$)

$$J_{H_2O_2} = J_{abs} \times \eta_{bulk} \quad (2)$$

Hence $\eta_{surface} = J_{H_2O}/J_{H_2O_2}$ (3)

And $\eta_{bulk} = J_{H_2O_2}/J_{abs}$ (4)

Here, $J_{H_2O_2}$ is the measured photocurrent density in 1 M NaOH with 0.5 M H_2O_2 and J_{abs} is the theoretical photocurrent density that the absorbed photons are totally converted. J_{abs} can be calculated according to the correlation between absorbance and irradiation:

$$P_d = P_0 10^{-A} \quad (5)$$

$$P_{abs} = P_0(1 - 10^{-A}) \quad (6)$$

P_0 (unit: mW cm^{-2} nm^{-1}) is power provided by solar simulator (in this case, AM 1.5G), P_{abs} is power of light actually absorbed by photoanode and P_d is power of light not absorbed at the photoanode (dissipated by reflection and penetration). A is absorbance of photoanode and light harvesting efficiency (LHE) is defined as $1 - 10^{-A}$. So the light not absorbed at the photoanode will be 10^{-A}. Integration of P_{abs}(λ) (mW cm^{-2} nm^{-1}) along with wavelength λ gives the total power density (unit of mW cm^{-2}), which is the

maximum power of photoanode.. The following formula shows the power of light absorbed by photoanode (J_{abs}):

$$J_{abs} = \int_{\lambda_1}^{\lambda_2} \frac{\lambda}{1240} P_{abs(\lambda)} d\lambda \left(\frac{mW}{cm^2}\right) \quad (7)$$
